# Proteomic profiling of maize opaque endosperm mutants reveals selective accumulation of lysine-enriched proteins

**DOI:** 10.1093/jxb/erv532

**Published:** 2015-12-27

**Authors:** Kyla J. Morton, Shangang Jia, Chi Zhang, David R. Holding

**Affiliations:** ^1^Department of Agronomy and Horticulture, Center for Plant Science Innovation, Beadle Center for Biotechnology, 1901 Vine Street, PO Box 880665, University of Nebraska, Lincoln, NE 68588-0665, USA; ^2^School of Biological Sciences, Center for Plant Science Innovation, Beadle Center for Biotechnology, 1901 Vine Street, PO Box 880665, University of Nebraska,Lincoln, NE 68588-0665,USA

**Keywords:** Endosperm, maize, non-zein, opaque, proteome, stress.

## Abstract

Reduced prolamin (zein) accumulation and defective endoplasmic reticulum (ER) body formation occurs in maize opaque endosperm mutants *opaque2* (*o2*), *floury2* (*fl2*), defective endosperm*B30 (*DeB30*), and *Mucronate* (*Mc*), whereas other opaque mutants such as *opaque1* (*o1*) and *floury1* (*fl1*) are normal in these regards. This suggests that other factors contribute to kernel texture. A liquid chromatography approach coupled with tandem mass spectrometry (LC-MS/MS) proteomics was used to compare non-zein proteins of nearly isogenic opaque endosperm mutants. In total, 2762 proteins were identified that were enriched for biological processes such as protein transport and folding, amino acid biosynthesis, and proteolysis. Principal component analysis and pathway enrichment suggested that the mutants partitioned into three groups: (i) *Mc*, *DeB30*, *fl2* and *o2*; (ii) *o1*; and (iii) *fl1*. Indicator species analysis revealed mutant-specific proteins, and highlighted ER secretory pathway components that were enriched in selected groups of mutants. The most significantly changed proteins were related to stress or defense and zein partitioning into the soluble fraction for *Mc*, *DeB30*, *o1*, and *fl1* specifically. *In silico* dissection of the most significantly changed proteins revealed novel qualitative changes in lysine abundance contributing to the overall lysine increase and the nutritional rebalancing of the *o2* and *fl2* endosperm.

## Highlight

Proteome-wide assessment across an isogenic collection of cloned opaque endosperm mutants caused by different and well-characterized mutations provided biological insight into proteome rebalancing and favorable amino acid compositional changes.

## Introduction

Maize endosperm differentiates into six distinct regions including the aleurone, subaleurone, embryo surrounding region, endosperm transfer cells, and central starchy region (Olsen *et al.*, 1999). The main endosperm storage tissues are the aleurone, subaleurone, and central starchy endosperm region. Aleurone cells accumulate mostly lipid bodies and protein storage vacuoles, while the starchy endosperm contains starch and endoplasmic reticulum (ER) protein bodies (Brouns *et al.*, 2012; Xiong *et al.*, 2013). The protein bodies are composed of zein polypeptides classified as α-, β-, γ-, and δ-zein (Lending and Larkins, 1989). ER-localized proteins bodies have an α- and δ-zein core surrounded by cross-linked γ-zein (Lending and Larkins, 1989; Woo *et al.*, 2001). For proper protein body formation, and the resulting vitreous endosperm, all zein classes must be present and in correct stoichiometric ratios (Guo *et al.*, 2013). Mutations affecting zein synthesis will not only alter protein body shape and size but also influence endosperm texture and cause opacity (Mertz *et al.*, 1964; Soave and Salamini, 1984; Schmidt *et al.*, 1990; Coleman *et al.*, 1997; Gillikin *et al.*, 1997; Kim *et al.*, 2003, 2006; Holding and Larkins, 2006; Holding *et al.*, 2007; Wang *et al.*, 2012; Holding, 2014).

Nearly isogenic lines of six opaque mutants were generated in the W64A inbred line comprising *opaque1* (*o1*), *opaque2* (*o2*), *floury1* (*fl1*), *floury2* (*fl2*), *Defective endosperm B30* (*DeB30*), and *Mucronate* (*Mc*) (Hunter *et al.*, 2002). All six mutants have been molecularly characterized and the majority of the mutations either directly or indirectly affect zein synthesis. *O2* encodes a bZIP transcription factor that regulates many genes including α-zeins (Schmidt *et al.*, 1990). *O2* also positively regulates the cytosolic pyruvate orthophosphate dikinase-1, which may influence starch to protein balance during endosperm development (Maddaloni *et al.*, 1996; Manicacci *et al.*, 2009). *o2* also has an almost 2-fold increase in the essential amino acids lysine and tryptophan due to proteome rebalancing (Schmidt *et al.*, 1990). *fl2*, *Mc* and *DeB30* are dominantly acting mutations in zein genes themselves and result in the accumulation of defective 22kDa α-zein, 16kDa γ-zein and 19kDa α-zein species, respectively (Coleman *et al.*, 1997; Kim *et al.*, 2003, 2006). In *fl2* and *DeB30*, point mutations result in uncleaved signal peptides that inappropriately anchor the dominant negatively acting zein polypeptide to the ER membrane, preventing movement into the ER lumen (Coleman *et al.*, 1997; Kim *et al.*, 2003). *Mc* has a 38bp deletion resulting in a nonsense, frameshift mutation (Kim *et al.*, 2006) and 63 abnormal amino acids on the C-terminal end of the protein. This reduces the interaction with the 22kDa α-zeins (Kim *et al.*, 2006). *DeB30*, *fl2*, and *Mc* result in increased expression of genes associated with an unfolded protein response (UPR), most likely due to the misshapen protein bodies and a disorganized ER lumen (Zhang and Boston, 1992; Shank *et al.*, 2001).

Opaque endosperm mutants that show little quantitative or qualitative differences in zein protein accumulation include *fl1* and *o1*. *FL1* encodes an ER membrane protein that is necessary for correct α-zein placement with the protein body core (Holding *et al.*, 2007). *O1* encodes a myosin XI protein that affects ER morphology and trafficking (Wang *et al.*, 2012). Interestingly, Fl1 has a domain (DUF593) that has been shown to function as a myosin XI receptor (Peremyslov *et al.*, 2013). This may suggest that Fl1 functions to attach protein bodies to the cytoskeleton. Although it has not been demonstrated, a direct or indirect physical or functional interaction between the O1 and Fl1 proteins is an interesting possibility.

Previous experiments using developing endosperms of *o1*, *o2*, *fl2*, and *Mc* mutants using an Affymetrix Gene Chip revealed that opaque mutants have diverse pleiotropic changes in gene expression (Hunter *et al.*, 2002). The global effect of gene expression appeared to correlate with the differences observed in changes of protein and amino acid synthesis. For example, *o1*, which has the least effect on zeins and amino acid composition, also has the least effect on global gene patterning. In contrast, *o2*, which has the largest reduction of zeins and subsequent increase in non-zeins, has profound changes in amino acid composition and the largest effect on global gene expression (Hunter *et al.*, 2002). Therefore, three phenotypic groups emerged: (i) W64A wild type (WT) and *o1*; (ii) *fl2* and *Mc*; and (iii) *o2* (Hunter *et al.*, 2002). Had *DeB30* been included in this experiment, it would likely have clustered with *fl2* and *Mc* due to a similar type of dominantly acting signal peptide mutation as *fl2*, a similar effect of lowered zein synthesis, and a common UPR induction. The UPR in *fl2*, *Mc*, and *DeB30* has been hypothesized to occur either directly because of the accumulation of abnormal zeins or because of non-zein proteins, which, by accumulating as a compensatory mechanism to rebalance the proteome, could overload the downstream ER secretory pathway (Hunter *et al.*, 2002; Guo *et al.*, 2012). The cause of endosperm stress in mutants such as *o1* and *fl1*, which have little effect on zein or non-zein accumulation, is unknown. It was suggested that any initiation of cellular stress requires substantial energy in the form of ATP, which in turn may become limiting in the developing endosperm of opaque mutants, leading to disruption of normal growth and, ultimately, opacity (Guo *et al.*, 2012). In support of this suggestion, the elevated expression levels of a number of heat-shock proteins (HSPs) observed in *o2* endosperm was shown to be returned to normal in modified *o2* (quality protein maize) endosperm (Guo *et al.*, 2012).

The opaque mutants have been well characterized, and in most cases a good explanation for the effects on endosperm texture has been provided. However, questions remain about the possible existence of a common underlying mechanism that could be shared across all, or a subset of, opaque endosperm mutants. Such a common mechanism may lead to the disruption of vitreous endosperm formation and could consequently explain the opaque phenotype in mutants for which only small quantitative or qualitative changes in zeins exist.

To understand the factors besides zeins that influence endosperm texture, a shotgun [liquid chromatography approach coupled with tandem mass spectrometry (LC-MS/MS)] proteome analysis of the non-zein fraction of nearly isogenic lines of six opaque mutants (*o1*, *o2*, *fl1*, *fl2*, *DeB30*,and *Mc*) together with W64A WT was performed. Previous experiments that investigated the transcriptome of selected opaque mutants (*o1*, *o2*, *fl2*, and *Mc*) can only be used to speculate about protein abundances. Key increased transcripts in *o2* include sorbitol dehydrogenase and glyceraldehyde 3-phosphate dehydrogenase genes, which both encode lysine-rich proteins (Jia *et al.*, 2013). Increased transcript expression of starch structural enzymes increased the amylopectin branching pattern in *o2* (Gibbon *et al.*, 2003; Jia *et al.*, 2013). The increased branching pattern may affect starch grain interaction with endosperm body proteins, thus promoting the opaque endosperm phenotype (Gibbon *et al.*, 2003; Jia *et al.*, 2013).

Multiple transcriptomic and two-dimensional SDS-PAGE experiments have been conducted on *o2* because of its value for studying proteome rebalancing and because of its importance as a high-lysine grain (Hunter *et al.*, 2002; Jia *et al.*, 2007; Frizzi *et al.*, 2010; Hartings *et al.*, 2011). Two-dimensional SDS-PAGE is labor intensive and expensive and is consequently not suitable for whole-proteome analysis. To assess the whole non-zein proteome, isogenic opaque endosperm mutants were analyzed using a label-free shotgun proteomic approach that was not biased towards selected abundant proteins or genetic background. The proteome of the non-zein fraction of each mutant was compared pairwise with WT and also with each other in order to identify factors that influence or result from the opaque kernel phenotype. The whole ER secretory pathway was found to be affected, in different areas, for each opaque mutant, which may be the cause of general cellular stress. There was a nearly 2-fold increase of lysine in *o2*, which was not only due to the quantitative increase of non-zein proteins but also to the qualitative enrichment of lysine in the most abundant proteins. This qualitative change in lysine-enriched proteins may also partly explain the lysine increase in opaque mutants lacking proteome rebalancing.

## Materials and methods

### Maize plant material

Nearly isogenic lines for maize opaque mutants were developed at the University of Arizona, and maintained at the University of Nebraska-Lincoln. Plants used for proteomic analysis were grown in Sunshine MVP (formerly Metro-Mix 200) soil under 16h day length cycles. Plants were grown in day temperatures ranging from 27 to 29 °C and night temperatures between 21 and 23 °C. The plants were self-pollinated, and developing kernels were harvested by plunge freezing whole ears in liquid nitrogen at 20 d after pollination.

### Zein and non-zein fractionation and SDS-PAGE analysis

For mutants and W64A WT, four biological replicate ears were run. Three whole kernels were taken from each ear and fractionated into alcohol-soluble zeins and aqueous non-zeins (Wallace *et al.*, 1990). Zein and non-zein fractions were checked using SDS-PAGE to make sure all samples had discrete, non-smeared bands indicating a lack of protein degradation. Total protein was quantified by a BCA colorimetric assay. Non-zeins were suspended in 8M urea with 50mM Tris/HCl (pH 8.0) and shipped on dry ice to the University of California-Davis Proteomic Core for in-gel trypsin digest and proteome evaluation by LC-MS/MS.

### Sample preparation and trypsin digestion

Protein samples were precipitated according to the manufacturer’s protocol using a ProteoExtract Protein Precipitation kit (CalBiochem). The resulting pellet was solubilized in 100 µl of 6M urea in 50mM ammonium bicarbonate. DTT (200mM) was added to a final concentration of 5mM and samples were incubated for 30min at 37 °C. Next, 20mM iodoacetamide was added to a final concentration of 15mM and incubated for 30min at room temperature, followed by the addition of 20 µl DTT to quench the iodoacetamide reaction. Lys-C/trypsin (Promega) was next added in a 1:25 ratio (enzyme:protein) and incubated at 37 °C for 4h. Samples were then diluted to <1M urea by the addition of 50mM ammonium bicarbonate and digested overnight at 37 °C. The following day, samples were desalted using C18 Macro Spin columns (Nest Group) and dried down by vacuum centrifugation.

### LC-MS/MS analysis

LC separation was done on a Waters Nano Acquity UHPLC (Waters Corporation) with a Proxeon nanospray source. The digested peptides were reconstituted in 2% acetonitrile/0.1% trifluoroacetic acid, and 3 µg of each sample was loaded onto a 100 μm×25mm Magic C18 100 Å 5U reverse-phase trap. The digested peptides were desalted online before being separated on a 75 μm×150mm Magic C18 200 Å 3U reverse-phase column. Peptides were eluted using a gradient of 0.1% formic acid (A) and 100% acetonitrile (B) with a flow rate of 300 nl min^–1^. A 60min gradient was run with 5 to 35% B over 100min, 35 to 80% B over 3min, 80% B for 1min, 80 to 5% B over 1min, and finally held at 5% B for 15min.

Mass spectra were collected on an Orbitrap Q Exactive Plus mass spectrometer (Thermo Fisher Scientific) in a data-dependent mode with one MS precursor scan followed by 15 MS/MS scans. A dynamic exclusion of 15s was used. MS spectra were acquired with a resolution of 70 000 and a target of 1×10^6^ ions or a maximum injection time of 30ms. MS/MS spectra were acquired with a resolution of 17 500 and a target of 5×10^4^ ions or a maximum injection time of 50ms. Peptide fragmentation was performed using higher-energy collision dissociation with a normalized collision energy value of 27. Unassigned charge states as well as +1 and ions greater than +5 were excluded from MS/MS fragmentation.

### Database searching

Tandem mass spectra were extracted and were analyzed using X! Tandem (The GPM, http://www.thegpm.org, accessed 10 December 2015; version CYCLONE 2013.02.01.1). X! Tandem was set up to search the Uniprot__20130710_VpC4HL database (124 576 entries assuming the digestion enzyme trypsin and the Uniprot__20140311_skARqM database (65822 entries). X! Tandem was searched with a fragment ion mass tolerance of 20 PPM and a parent ion tolerance of 20 PPM. Carbamidomethyl of cysteine was specified in X! Tandem as a fixed modification. Glu→pyro-Glu of the N terminus, ammonia loss of the N terminus, Gln→pyro-Glu of the N terminus, deamidation of asparagine and glutamine, oxidation of methionine and tryptophan, dioxidation of methionine and tryptophan, and acetylation of the N terminus were specified in X! Tandem as variable modifications.

#### Scaffold criteria for protein identification 

Scaffold (version Scaffold_4.4.1, Proteome Software, Portland, OR, USA) was used to validate MS/MS-based peptide and protein identifications. Peptide identifications were accepted if they could be established at >95.0% probability by the Scaffold local false discovery rate (FDR) algorithm resulting in an FDR of 0.4%. Protein identifications were accepted if they could be established at >99.0% probability and contained at least two identified peptides resulting in an FDR of 0.03%. Protein probabilities were assigned by the Protein Prophet algorithm (Nesvizhskii *et al.*, 2003). Proteins that contained similar peptides and could not be differentiated based on MS/MS analysis alone were grouped to satisfy the principles of parsimony. Proteins sharing significant peptide evidence were grouped into clusters. Proteins were annotated with gene ontology (GO) terms from gene association.goa_uniprot (downloaded 1 May, 2013) (Ashburner *et al.*, 2000).

#### Quantitative data normalization and proteomic fold-change analysis 

Protein abundance data were calculated by Scaffold software based upon the normalized spectral abundance factor (NSAF) method (Zybailov *et al.*, 2006). A spectral fraction of 0.05 was initially added to all values to compensate for a spectral reading of null or zero and to allow for log transformation of the raw data file (Mirzaei *et al.*, 2012). The NSAF method determines abundance based upon the number of exclusive spectra (*SpC*) divided by the amino acid length (*L*) of the protein and then normalizes based upon the sum of *SpC/L* for all the proteins experiment-wide. The averages of the biological replicates for NSAF values were used for statistical analysis of protein abundance. Several pairwise *t*-tests were performed in Scaffold between each mutant and WT to find proteins of significant differential abundance. Significant protein differences were assigned if the *P* value was <0.01. Fold change was also calculated on normalized values and was filtered by a fold change >2 or <0.5.

#### Amino acid content analysis 

Trypsin-digested peptide fragmented counts measured by mass spectrometry were employed for amino acid content analysis. The trypsin-digested peptide fragmented counts were used for pairwise comparisons among genotypes and significantly differentially expressed proteins were identified by the R package DESeq2 (Love *et al.*, 2014). All proteins were sorted by their *P* values, and the top-ranked proteins were kept for amino acid content calculation. For a comparison between one mutant and WT, top-ranked proteins were split into two groups, increased or decreased proteins. To estimate the individual amino acid contents, the frequency of a given residue was counted in selected top-ranked proteins, and the percentage content of each amino acid residue was the ratio of its frequency to the total length of selected top-ranked protein sequences. The ratio of percentage content in increased proteins to decreased proteins for a given type of amino acid was calculated before log_2_ calculations. Furthermore, we conducted a more detailed analysis for lysine increase by incorporating protein abundance. The contribution of a top-ranked selected protein to lysine increase in a mutant was evaluated by two factors: first, lysine content level against the average, and secondly, changed protein abundance between WT and mutant. The abundance data was normalized based on the total abundance in a mutant. The protein sequences were obtained from the UniProt Database (http://www.uniprot.org/uploadlists/, accessed 10 December 2015).

#### Functional annotation clustering and GO enrichment analysis 

To gain insight into the biological significance of enriched protein clusters, the Database for Annotation, Visualization, and Integrated Discovery (DAVID, http://david.abcc.ncifcrf.gov, accessed 10 December 2015) was used. DAVID provides batch annotations to highlight the most relevant GO terms associated with a query protein list (Huang *et al.*, 2008, 2009). Functional annotation clustering analysis in DAVID was used to identify which annotation groups were enriched in the total proteins identified in the non-zein maize endosperm fraction. DAVID generates an enrichment score for a group of genes indicating annotation term member associations in a given experiment. The enrichment score is the negative log scale of the geometric mean of each member’s Fisher’s exact *P* value transformed into an EASE score within the annotation cluster. An enrichment score of 1.3 is equivalent to a non-log scale *P* value of 0.05.

To identify pathways specifically enriched compared with WT for each mutant, a bin-wise Wilcoxon test in PageMan (Usadel *et al.*, 2006) was conducted. Enriched proteins were considered to be over-represented when associated with a *P* value <0.05. This statistical test is similar to a rank-based *t*-test and tests if the median NSAF fold-change value of a protein(s) is the same as other median fold-change values of all bins within the ontological group. No multiple test correction was applied to the dataset using the mapping file “Zm_B73_5b_FGS_cds_2012”.

#### Plotting and statistical analysis 

Principal component, indicator species, clustering, and PageMan (Usadel *et al.*, 2006) analyses were applied to the proteomic data. For principal component analysis (PCA), the protein abundance data was used as a vector for a given maize mutant, and the R functions *prcomp*() and *ggbiplot*() were employed for data analysis and plotting, respectively. Enriched proteins were identified using indicator species analysis of the relationship between the protein abundance values from all samples. The function *multipatt*() in the R package *indicspecies* was employed to conducted indicator species analysis, and the association index employed an unequal-group-corrected point biserial correlation coefficient (De Cáceres *et al.*, 2012). Abundance levels of some proteins in mutants were presented as a heatmap plot, which was generated by the function of *heatmap.2*() (R package: *gplots*), and dendrograms were plotted based on the hierarchical clustering function, *hclust*(), in R with the Pearson correlation distance and average linkage method.

### RNA-sequencing (RNA-seq) analysis

Plant material preparation, RNA preparation, library construction, Illumina RNA-seq using a Genome Analyzer II platform (Illumina) and data analysis were carried out according to a previously published work (Guo *et al.*, 2012).

### Accession codes

The mass spectrometry proteomic data have been deposited into the ProteomeXchange Consortium (http://proteomecentral.proteomexchange.org, accessed 10 December 2015) via the PRIDE partner repository (Vizcaíno *et al.*, 2013) with the dataset identifier PXD002379.

## Results

### Overview of the analysis of the total non-zein proteome

**Fig. 1.**
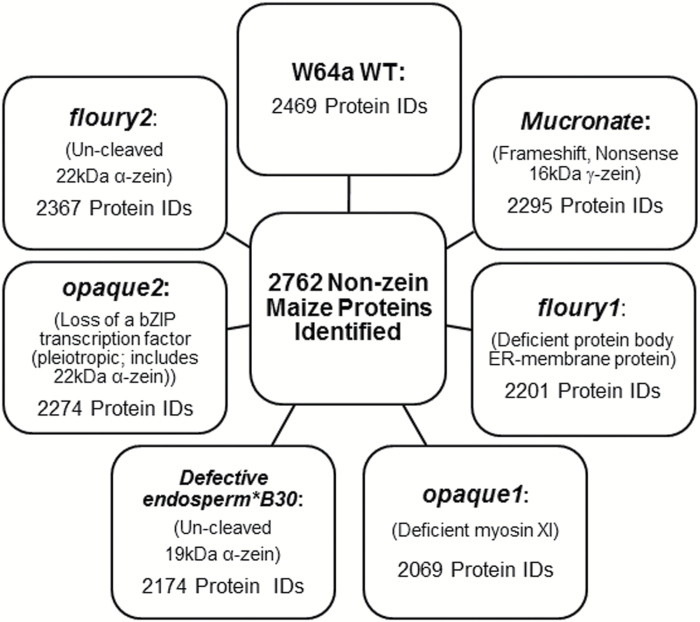
Total proteins identified in all opaque mutants. A total of 2762 proteins were identified by LC-MS/MS in the non-zein fraction of the maize endosperm. The number of proteins identified in each sample is shown and the molecular basis of the mutation for each opaque is given for reference. W64A WT had the most identifiable proteins followed by *fl2*, *Mc*, *o2*, *fl1*, *DeB30*, and *o1*.

To compare the abundance of non-zein proteins in opaque endosperm mutants, a shotgun proteomics approach was performed. The non-zein fraction from each opaque mutant and WT was separated by liquid chromatography coupled to tandem mass spectrophotometry. A total of 2762 proteins were confidently identified across all endosperm samples and corresponding biological replications (Fig. 1). For each mutant the number of proteins identified ranged from 2069 to 2469 proteins (see Supplementary Table S1 at *JXB* online). WT had the highest number of proteins identified and all mutants had a smaller non-zein proteome. *fl2*, *Mc*, and *o2* had over 90% of the WT number of proteins identified, while *fl1* and *DeB30* had just less than 90%. *o1* had the lowest number of proteins identified at 84% of WT.

### Proteome rebalancing in *o2* and *fl2* and its impact on lysine content

**Fig. 2.**
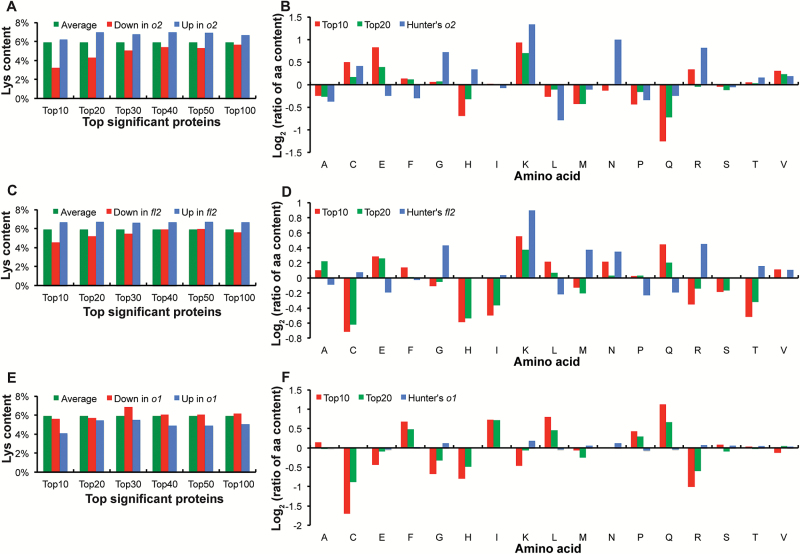
Amino acid content analysis in non-zein proteins for *o2*, *fl2*, and *o1* compared with WT. (A, C, E). Lysine (K) content was calculated for the top significant increased and decreased proteins in *o2* (A), *fl2* (C), and *o1* (E), compared with WT. The average lysine content of the most increased (Up, blue) and decreased (Down, red) proteins, and all proteins (Average, green) were compared from the top 10 to the top 100. (B, D, F) The amino acid content data (blue, in percentage protein, w/w) in *o2* (B), *fl2* (D), and *o1* (F) from Hunter (2002). Amino acid abbreviations are shown in alphabetical order, and Glx from Hunter (2002) was placed on both Glu (E) and Gln (Q).

Compared with WT, an increase in the ratio of non-zeins to zeins in *o2* and *fl2* results in increased lysine content, along with other amino acid composition changes, but no significant lysine increase is found in *o1* (Hunter *et al.*, 2002). The global quantitative non-zein abundance therefore plays an important role in nutritional rebalancing in *o2* and *fl2*. However, protein compositional changes may also contribute to the final lysine increase, in terms of both up-regulated lysine-enriched proteins and down-regulated lysine-poor proteins. To address the relative contributions of the global non-zein increase and the lysine content of individual proteins to overall lysine increase, the lysine content of the most significantly accumulating proteins in mutants and WT were compared. Since the comparison of non-zeins was on a ‘same protein weight’ basis between mutant and WT, this *in silico* comparison excluded the overall quantitative increase of non-zeins for proteome rebalancing mutants. These analyses showed that, compared with the overall average lysine content of all proteins in the UniProt Database (5.91%), the lysine content in *o2* was enriched in the most proteins with increased abundance and reduced in the most proteins with decreased abundance, ranked according to *P* value. This indicated that more lysine-enriched proteins were increased (Fig. 2A, blue bars), and the lysine content ratio of top up/down proteins furthermore confirms a qualitative lysine increase in *o2* (Fig. 2B, red and green bars). The identities of the top 30 most abundant proteins that are increased in *o2*, as well as the top 30 most abundant proteins that are decreased in *o2* with respect to WT, along with their individual lysine content and relative abundance are shown in Supplementary Table S2 at *JXB* online.

*fl2* showed a similar pattern of a qualitative increase in lysine content to *o2* (Fig. 2C, D), while *o1* showed the opposite (Fig. 2E, F) with almost no changes of the top 20 proteins. The lysine enrichment was also clearly shown by the relatively lower lysine content of proteins that were decreased in *o2* and *fl2* (Fig. 2A, C, red bars). As shown for *o2*, the identities of the top 30 most abundant proteins that were increased in *fl2* and *o1*, as well as the top 30 most abundant proteins that were decreased in *fl2* and *o1* with respect to WT, along with their fold changes and individual lysine content are shown in Supplementary Table S2. In *o2* and *fl2*, the trends for an increase in lysine-rich proteins (Fig. 2A, C, blue bars) and the more prominent trend for a reduction in lower lysine content proteins (Fig. 2A, C, red bars) diminished as the number of top most abundant proteins increased towards 100 and their overall abundance contributed less to the total protein.

The fact that *o1* did not show this effect (Fig. 2E, blue and red bars) is consistent with the observation that this mutant does not have increased lysine. Hunter *et al.* (2002) determined the overall amino acid compositions (w/w) in total endosperm protein of the same isogenic opaque mutants, which included zein amino acid contributions. However, the present *in silico* analysis performed on the generated proteomic data was selective to the non-zein endosperm proteome. The comparison with previous research allowed assessment of the qualitative contributions of non-zein proteins to the overall lysine and tryptophan content within the endosperm, since these amino acids are missing from zein polypeptides. The results showed that the calculated lysine content of the most abundant non-zein proteins in *o2* and *fl2* was consistent with the previously measured lysine increase (Hunter *et al.*, 2002), compared with WT, while in *o1*, the calculated lysine content was opposite from the measured content (Fig. 2B, D, F).

The amino acid content ratio comparisons with the previously measured amino acid content (Hunter *et al.*, 2002) were extended to include other amino acids. In Fig. 2B, besides Lys (K), Cys (C) and Val (V) with positive values of log_2_ ratios of up/down amino acid content, and Ala (A), Leu (L), Met (M), Pro (P), and Gln (Q) with negative values, are consistent with previous data (Hunter *et al.*, 2002). However, with the exception of lysine, the same trends were not seen for other amino acids in *fl2* (Fig. 2D). This may mean that the qualitative lysine enrichment in the top proteins in *fl2* contributes more significantly to the overall lysine increase than the global quantitative increase in non-zein proteins. Interestingly, there were almost no amino acid content changes in *o1* compared with the results of Hunter *et al.* (2002), although our analysis of non-zein amino acid content showed changes (Fig. 2F). This observation indicated that our lysine content analysis agreed with previously measured overall seed lysine content in *o2* and *fl2* and the lack thereof in *o1*. Lysine content was also increased in *Mc* and *DeB30* but not in *fl1* (see Supplementary Fig. S1 at *JXB* online).

In addition to the relative change in a protein between WT and mutant (fold change) and the calculated lysine content, it is also important to take into account the abundance of that protein relative to other proteins in the proteome. A highly abundant, lysine-rich protein with a 2-fold increase is expected make a greater impact on seed lysine content than a similarly changed protein of low abundance. Within the top 30 most significantly changed proteins based on *P* value, Table 1 combines lysine content, change in a given protein between WT and mutant, and the relative (normalized) abundance of that protein. After being normalized based on the total abundance, the abundance difference was compared between mutants and WT to assess global non-zein proteomic changes. Top proteins with relatively high lysine contribution were identified as either increased and lysine-enriched or decreased and having below-average lysine in *o2* and *fl2* (Table 1). Although not universal, in general, identified proteins showed both abundance and lysine content changes in the same direction (Supplementary Table S2). The lysine contribution of the top proteins in the *o1* non-zein proteome was much smaller compared with *o2* and *fl2*. These lysine contribution results confirmed the above lysine content results for the three mutants in Fig. 2, and suggested that the qualitative protein composition change also plays an important role in lysine increase, in addition to the previously identified quantitative non-zein increase in *o2*.

**Table 1.** Top contributing proteins for qualitative lysine increase in opaque endosperm mutants

	**UniProt ID**	**Description**	***P* value** ^***a***^	**Lys** ^***b***^	**Norm_WT** ^***c***^	**Norm_MU** ^***c***^	**Con** ^***d***^
Up_*o2* ^*e*^	C5XX52	Glyceraldehyde-3-phosphate dehydrogenase	1.17E–05	8.31%	1434.91	3561.00	51.03
	B6SHW9	Ubiquitin fusion protein	8.13E–04	13.18%	307.84	549.44	17.56
	B4FAL9	Fructose-bisphosphate aldolase	9.88E–06	8.45%	853.65	1313.69	11.68
	B4FT23	14-3-3-like protein	1.05E–05	7.54%	934.66	1551.91	10.06
	C0PHR4	Adenosylhomocysteinase	1.69E–04	7.63%	393.92	933.63	9.28
Down_*o2* ^*e*^	B6SIX6	Prolamin PPROL 17	6.02E–09	0.56%	198.48	6.89	10.25
	P04698	Zein-α PZ22.3	2.99E–10	0.37%	192.40	23.41	9.36
	B6SI09	Aquaporin TIP3.1	5.57E–06	1.12%	229.87	53.70	8.44
	B6SJ53	*22kDa α-zein* ^*f*^	3.00E–07	0.38%	95.19	5.51	4.96
	B6TIK6	Sarcosine oxidase	4.02E–11	3.13%	179.24	24.79	4.29
Up_*fl2* ^*e*^	B6TNF1	Calnexin	5.01E–19	11.61%	91.14	504.55	23.56
	P24067	Luminal-binding protein 2	1.73E–16	9.35%	325.06	910.99	20.16
	A5A5E7	Protein disulfide isomerase	1.13E–05	9.36%	930.61	1512.57	20.08
	B4FT23	14-3-3-like protein	1.81E–07	7.54%	934.66	1624.69	11.25
	Q5EUD5	Protein disulfide isomerase	4.24E–21	8.66%	89.11	448.49	9.88
Down_*fl2* ^*e*^	Q09HU3	Trypsin inhibitor (fragment)	7.18E–11	1.57%	747.33	141.23	26.30
	Q946V2	Legumin 1	5.14E–12	2.48%	1094.66	416.15	23.27
	Q43706	Sucrose synthase	6.57E–07	5.15%	1581.74	635.00	7.20
	B6TIK6	Sarcosine oxidase	1.71E–15	3.13%	179.24	1.08	4.95
	C0LNQ9	UDP-glucosyltransferase	1.48E–06	2.34%	179.24	60.37	4.24

^*a*^ *P* value is determined by doing pairwise comparisons between one genotype and WT in DESeq2.

^*b*^Lysine content of each protein was calculated based on the downloaded protein sequences from the UniProt Database.

^*c*^Norm_WT and Norm_MU are for the normalized abundances for WT, *o2*, and *fl2*, using raw abundance divided by the total abundance in one sample.

^*d*^Contribution to lysine increase was calculated for one specific protein by using two factors: normalized abundance difference between mutant and WT, and lysine content difference against the average (5.91%).

^***e***^The up-regulated and down-regulated proteins in *o2* and *fl2* are labeled as “Up” and “Down”, respectively, compared with WT.

^*f*^An italicized protein name indicates an uncharacterized protein, which was annotated with significant a BLASTP homology search identifier.

Although post-translational factors play a major role in the abundance of proteins and vary widely between different proteins, regulation at the transcriptional and RNA stability levels also plays a major role in the eventual abundance. We were interested to explore the extent of this correlation in developing maize seeds. Using RNA-seq data that compared the same developmental seed stage of W64a WT and W64a *o2* (Guo *et al.*, 2012), we mined the abundance values for transcripts of the same top 30 proteins increased and decreased in *o2*. The read numbers, fold change, and *P* values are shown in Table S2 to the right of the protein abundance and lysine content values. In general, these results showed a strong correlation between protein and transcript abundance for proteins both increased and decreased in *o2*. The magnitude of change between a given protein and transcript was not directly comparable, but it was striking that the trend was very often in the same direction. These results supported the assertion that conclusions on quantitative abundance of proteins can be drawn from the proteomics results.

### Functional annotation clustering and pathway enrichment analysis

A large number of proteins in the non-zein fraction were identified. To determine which proteins were significantly enriched, a method of functional annotation clustering provided by the DAVID bioinformatics institute was used (Huang *et al.*, 2009). Listed in Table 2 are the top 10 functional annotation clusters for total non-zein proteins ranked by the assigned enrichment score. The enrichment score is the negative log scale of geometric mean of each member’s Fisher’s exact *P* value transformed into an overall score for each annotation cluster. This enrichment tool was selective to proteins that have been annotated or for which domain-of-function information is available. Out of the 2762 proteins, 1258 could be mapped to DAVID protein IDs. The most significant cluster contained proteins with RNA recognition or binding motifs. Typically, proteins with RNA recognition motif domains are ribonucleoproteins (ribosomes), transcription factors, and spliceosomes (Mitchell *et al.*, 2015). Importantly, ribonucleoproteins were also listed as an independent cluster in the top 10 ranked at seven. Cellular component enrichment was prevalent for organelle and mitochondrial membranes. The most enriched biological processes included protein transport, protein folding, proteolysis, and biosynthetic processes involving nitrogen or organic compounds.

**Table 2.** Ten highest functional annotation clusters for all proteins identified in the maize non-zein endosperm fraction

	**GO category or database**	**Functional annotation term**	**Protein count**	***P* value**
Annotation cluster 1 enrichment score: 4.47	SMART	RNA recognition motif	35	2.50E–06
	INTERPRO	Nucleotide-binding, α-β plait	35	1.00E–04
	INTERPRO	RNA recognition motif, RNP-1	35	1.50E–04
Annotation cluster 2 enrichment score: 4	Biological Process	Intracellular transport	28	4.30E–06
	Biological Process	Intracellular protein transport	24	9.20E–06
	Biological Process	Cellular protein localization	24	1.60E–05
	Biological Process	Cellular macromolecule localization	24	1.60E–05
	Biological Process	Protein transport	34	1.80E–03
	Biological Process	Establishment of protein localization	34	1.80E–03
	Biological Process	Protein localization	34	3.20E–03
Annotation cluster 3 enrichment score: 3.5	Cellular Component	Mitochondrial part	29	4.30E–07
	Cellular Component	Organelle envelope	26	5.60E–06
	Cellular Component	Envelope	26	9.00E–06
	Cellular Component	Organelle membrane	33	1.30E–05
	Cellular Component	Mitochondrial membrane	22	6.00E–05
	Cellular Component	Mitochondrial envelope	22	1.50E–04
	Protein Keyword	Mitochondrion inner membrane	7	3.80E–04
	Cellular Component	Mitochondrial inner membrane	16	3.30E–03
	Cellular Component	Organelle inner membrane	16	4.10E–03
Annotation cluster 4 enrichment score: 3.24	Biological Process	Proteolysis	65	2.10E–07
	Molecular Function	Peptidase activity	50	3.20E–04
	Molecular Function	Peptidase activity, acting on l-amino acid peptides	45	7.80E–04
	Protein Keyword	Protease	21	2.80E–02
	Molecular Function	Endopeptidase activity	27	4.30E–02
Annotation cluster 5 enrichment score: 3.15	Biological Process	Nitrogen compound biosynthetic process	48	3.60E–05
	Biological Process	Cellular amino acid biosynthetic process	22	1.10E–03
	Biological Process	Amine biosynthetic process	23	1.70E–03
	Biological Process	Organic acid biosynthetic process	32	1.70E–03
	Biological Process	Carboxylic acid biosynthetic process	32	1.70E–03
Annotation cluster 6 enrichment score: 3.13	Biological Process	Protein folding	29	8.10E–06
	Protein Keyword	Chaperone	15	3.30E–03
	Molecular Function	Unfolded protein binding	15	1.50E–02
Annotation cluster 7 enrichment score: 3.03	SMART	Sm	9	1.80E–04
	INTERPRO	Like-Sm ribonucleoprotein, eukaryotic and archaea-type	9	6.50E–04
	INTERPRO	Like-Sm ribonucleoprotein, core	9	8.80E–04
	Protein Keyword	viral nucleoprotein	8	7.60E–03
Annotation cluster 8 enrichment score: 2.91	INTERPRO	Universal stress protein A	8	2.00E–04
	INTERPRO	Rossmann-like α/β/α sandwich fold	14	2.20E–03
	INTERPRO	UspA	8	4.30E–03
Annotation cluster 9 enrichment score: 2.66	Biological Process	Proteolysis	65	2.10E–07
	Biological Process	Protein catabolic process	29	3.40E–05
	Biological Process	Cellular protein catabolic process	26	8.10E–05
	Biological Process	Proteolysis involved in cellular protein catabolic process	26	8.10E–05
	Biological Process	Macromolecule catabolic process	32	1.60E–04
	Biological Process	Cellular macromolecule catabolic process	26	1.80E–04
	Biological Process	Modification-dependent macromolecule catabolic process	22	5.10E–04
	Biological Process	Modification-dependent protein catabolic process	22	5.10E–04
	Protein Keyword	Ubl conjugation pathway	12	8.70E–04
	Protein Keyword	Ligase	19	2.70E–03
	SMART	UBCc	10	1.30E–02
	INTERPRO	Ubiquitin-conjugating enzyme, E2	10	3.70E–02
	INTERPRO	Ubiquitin-conjugating enzyme/RWD-like	10	4.10E–02
Annotation cluster 10 enrichment score: 2.48	Cellular Component	Membrane-enclosed lumen	15	3.40E–04
	Cellular Component	Mitochondrial matrix	7	1.20E–03
	Cellular Component	Mitochondrial lumen	7	1.20E–03
	Cellular Component	Intracellular organelle lumen	12	4.70E–03
	Cellular Component	Organelle lumen	12	4.70E–03

The enrichment score is the negative log scale of geometric mean of each member’s Fisher’s exact *P* value transformed into a score within the annotation cluster. An enrichment score of 1.3 is equivalent to a non-log scale *P* value of 0.05.

**Fig. 3.**
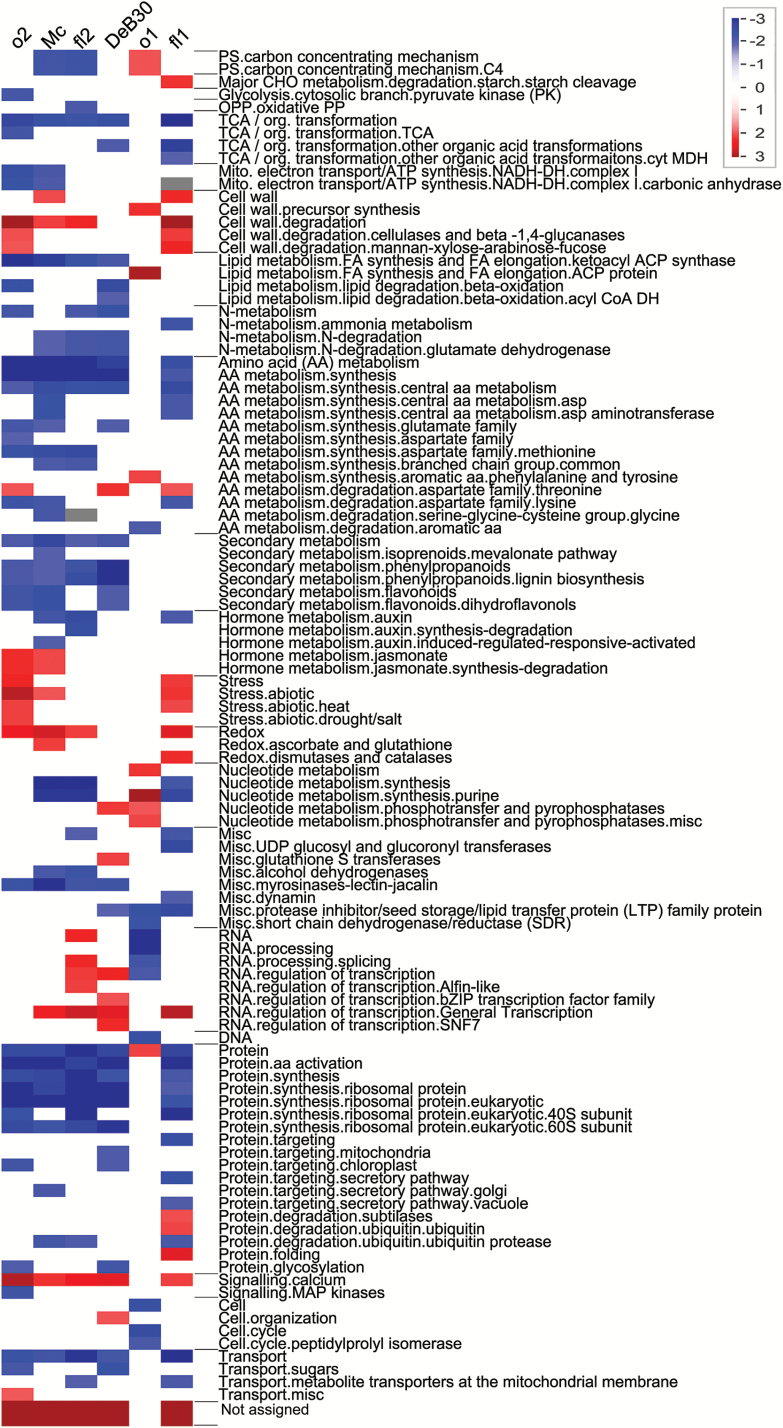
Protein over-representation analysis of opaque endosperm mutants. PageMan graphical visualization of enriched pathways for each opaque mutant and corresponding fold-change values compared with WT. Red boxes indicate increased abundance for proteins belonging to a specific function or annotation bin of the corresponding mutant, while blue indicates a lower abundance. Lines indicate upper and lower boundaries of each specific functional bin as determined by PageMan.

To identify the key pathways affected in the diverse set of opaque mutants, NSAF fold-change values (see Supplementary Table S7 at *JXB* online) were used compared with WT and were subjected to a Wilcoxon bin-wise statistical test as graphically represented in Fig. 3. Overall, 1489 of the total proteins identified experiment-wide could be correlated to gene identifiers and thus mapped. This proportion was similar to the DAVID GO cluster analysis. All mutants except *o1* had a decrease in general metabolism including nitrogen, amino acid, lipid, nucleotide, and other secondary pathways. A general increase was seen for cell wall degradation, stress and redox proteins, RNA regulation, and calcium signaling. The mutant with the least significant fold-change proteins compared with WT was *o1*. Possible increased metabolism pathways included fatty acid synthesis, aromatic amino acid, and nucleotide synthesis. Pathway enrichment revealed three groups of opaque mutants that had similar effects on metabolism and included *o2*, *Mc*, *fl2*, and *DeB30*, which was a considerable contrast to that of *fl1* and was even more pronounced compared with differences in *o1*.

### Indicator species enrichment analysis for grouping of opaque endosperm mutants

An indicator species analysis (ISA) was used to find proteins that were either unique to a specific mutant or were found to be enriched in differential grouping of mutants. In this analysis, a total of 1573 proteins were found to be present in at least one sample with a significant *P* value of <0.05. Twenty-five percent of the evaluated proteins were unique to a specific genotype. The most unique sets of proteins were identified in *o1* and *fl1* with 109 and 106 proteins, respectively (Table 3). In a previous study of opaque mutants (Hunter *et al.*, 2002), it was shown that the more pleiotropic the effects of the mutation, the more diverse the transcriptome. Here, *o1* and *fl1* mutants, which do not substantially affect zein accumulation, had the highest number of unique proteins. Other mutants with very few unique proteins included *Mc* and *DeB30*, while WT and *fl2* had intermediate numbers of unique proteins.

**Table 3.** Indicator species analysis ISA highlighted 1573 significant proteins (*P*<0.05) in at least one sample. Total proteins for each numbered group are given in parentheses.

**Unique protein enrichment**		**Two-group enrichment (574 proteins**)		**Three-group enrichment (282 proteins**)		**Four-group enrichment (197 proteins**)		**Five-group enrichment (110 proteins**)		**Six-group enrichment (23 proteins**)	
*o1*	109	WT+*o1*	378	*fl2+Mc+DeB30*	45	*Mc+fl1 +fl2+ DeB30*	64	*o2+Mc+fl1+fl2+DeB30*	42	WT*+o2+fl1+fl2+Mc+DeB30*	13
*fl1*	106	*fl1+fl2*	34	WT*+fl2+o1*	43	WT*+Mc+fl2+DeB30*	26	WT*+o2+Mc+fl2+DeB30*	32	α-soluble NSF attachment protein	
WT	73	*fl2+DeB30*	31	*fl1+fl2+DeB30*	30	*o2+Mc+fl2+DeB30*	16	WT*+Mc+fl2+DeB30+o1*	17	WT*+o2+o1+fl2+Mc+DeB30*	5
*fl2*	65	WT+*fl2*	26	WT*+fl1+o1*	29	WT*+Mc+fl2+o1*	15	WT*+Mc+fl1+fl2+DeB30*	7	Indole-3-acetate β-glucosyltransferase	
*DeB30*	23	*fl1+o1*	24	WT+*DeB30+o1*	20	WT*+fl2+DeB30+o1*	15	WT*+o2+fl2+DeB30+o1*	4	WT*+o1+fl1+fl2+Mc+DeB30*	3
*o2*	7	*fl2+Mc*	18	*Mc+fl1+fl2*	20	WT*+o2+Mc+ fl2*	10	WT*+Mc+fl1+fl2+o1*	3	Protein transport protein Sec61 β	
*Mc*	4	*fl1+DeB30*	10	WT+*Mc+fl2*	15	*o2+Mc+fl1+fl2*	10				

Of the 109 unique *o1* proteins, only 38 were functionally annotated, and no significant biological or molecular process information was available other than protein localization enrichment for the cytoplasm component. The top unique proteins in *o1* were hydroxymethylbutenyl 4-disphosphate synthase, glycine cleavage complex P-protein, ribonuclease 3, and a trehalose phosphatase/synthase family protein (see Supplementary Table S3 at *JXB* online). In contrast, the pleiotropic *o2* mutant had seven unique proteins, which were all defense or stress response related. The unique proteins with the highest association values with *o2* included disease resistance response protein 206, senescence-associated protein DIN1, and a low-molecular-weight cysteine-rich protein (LCR70). The highest associated protein unique to *Mc* was the *Mucronate*-specific 16kDa γ-zein frameshift protein and was also highlighted as one of the 50 most abundant proteins (Table 2). Unique proteins for *fl1* were enriched for negative regulators of molecular function and catalytic activity of subtilase family proteins. The top five unique proteins identified for *fl1* included purple acid phosphatase, two uncharacterized proteins, β-amylase, and a putative glyoxalase family protein. *fl2* unique proteins were mostly defense response related to herbicide and temperature but were enriched for glutathione and oligopeptide-binding motifs. Two proteins that had a higher than 90% association with *fl2* were HSP20 and HSP70. Other unique proteins included embryonic abundant protein 1 and α-6-galactosyltransferase. Unique proteins in *DeB30* were enriched for glutathionylation or the addition of glutathione to cysteine residues. In the top proteins specific to *DeB30* were multiple clusters of IN2-1 proteins whose transcripts are increased upon treatment with herbicide (Hershey and Stoner, 1991). It has been suggested previously that IN2-1 is involved in several plant stress situations and that their function is associated with safener-induced herbicide tolerance (Farago *et al.*, 1994).

The highest percentages of evaluated significant proteins were shared at the two-grouping enrichment level with 574 proteins. WT and *o1* shared the highest number of significant proteins with 378 in common with one another. These shared proteins were enriched for vitamin B6 and GTP binding and also for aminotransferase and peptidase activity but generally spanned across all metabolism pathways (see Supplementary Table S4 at *JXB* online).

The UPR opaque mutants (*fl2+Mc+DeB30*) had the highest number of shared proteins for the three-group enrichment, which mostly contained protein-folding and translation-related proteins. The top proteins shared in this grouping were a cluster of protein disulfide isomerase, an uncharacterized protein with a function of exocytosis, a KH domain-containing protein, a cluster of ribosomal proteins, and a mitochondrial fission protein (see Supplementary Table S5 at *JXB* online).

Six-group enrichment was useful because it showed proteins that were specifically enriched in all but one sample (see Supplementary Table S6 at *JXB* online). This grouping highlighted that whichever genotype was absent from the comparison had a differential abundance for a protein belonging to the specified grouping of the genotype. The top number of proteins shared in this complex grouping was WT*+o2+fl1+fl2+Mc+DeB30*, which included every opaque mutant except *o1*. One ER secretory protein identified in this grouping was an α-soluble *N*-ethylmaleimide-sensitive factor (NSF) attachment protein (α-SNAP), which is an intracellular membrane protein that functions to help reset or recycle vesicle transport machinery using ATP (Eakle *et al.*, 1988; Malhotra *et al.*, 1988). In the six grouping that excluded *o2* from enrichment, a central component of ER translocation, the Sec61-β protein, was a highly significant (*P*=0.008) protein. Through ISA six-group enrichment, *fl1* was identified as having a lower abundance for indole-3-acetate β-glucosyltransferase compared with all other genotypes. This protein is known to play a key role in plant hormone homeostasis and may indicate adverse hormone auxin signaling in the developing endosperm (Ostrowski and Jakubowska, 2014).

### PCA of non-zein proteome structure

**Fig. 4.**
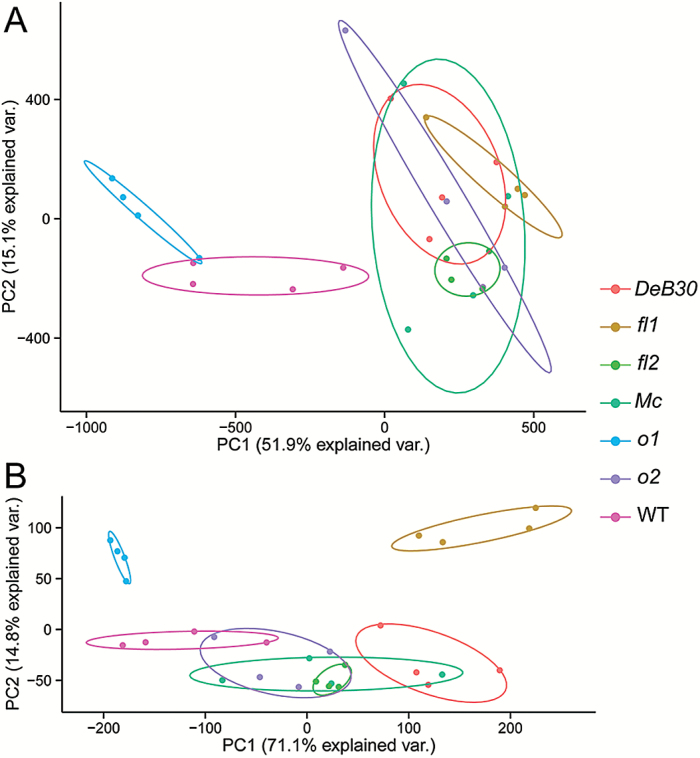
(A) PCA of all proteins identified experiment-wide. (B) PCA evaluating only proteins with significant fold changes (*P*<0.01; fold change >2 or <0.5) compared with W64A WT.

PCA was used to evaluate all the proteins identified to visualize the internal structure or structured variability across samples (Fig. 4A). Each biological replication for a given genotype was evaluated and their relationship denoted by a circle inclusion. The all-protein PCA could describe 51.9% of the explained variance in component 1 and 15.1% in component 2, totaling 67% of the explained variance. WT and *o1* could be separated spatially by component 1 from all other opaque mutants. This was also supported by the two-group enrichment ISA demonstrating a commonality between WT and *o1*. Component 2 highlighted the near separation of *o1* from WT with all other mutants but still demonstrated a substantial overlap. We observed that, in the top 30 proteins, few increased and decreased proteins were shared among *o2*, *fl2*, and *o1* (Supplementary Table S2). However, when evaluating all proteins, *Mc*, *o2*, and *DeB30* demonstrated strong variation across biological replicates.

This variation could be reduced when limiting the evaluated proteins to include only those with significant fold changes (*P*<0.01; fold change >2 or <0.5). Thus, a second PCA explained 85.9% of the variance for the selected significant proteins (Fig. 4B). In this PCA, *o1* and *fl1* were clearly separated from one another but also from all other samples. This indicated that the proteins that showed the greatest divergence from WT or normal levels were vastly different and unique to the opaque mutant. For *o2*, *fl2*, *Mc*, and *DeB30*, a greater separation could be seen using only significant proteins, but there was still substantial overlap.

### Top significant proteins with changed abundance in opaque mutants

**Fig. 5.**
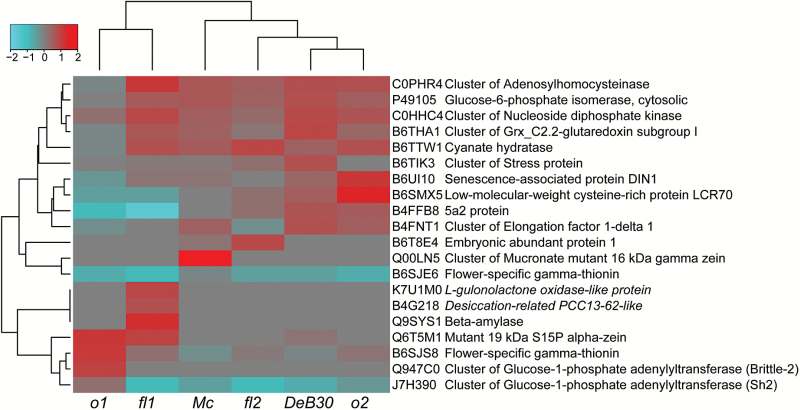
Heat map of significant fold-changed proteins in mutants compared with W64A WT. Proteins are color coded by fold-change ratio values compared with WT. Red boxes indicate increased abundance in the corresponding mutant, while green indicates a lower abundance in the mutant. The dynamic range was from 7.4 to 0.2 fold change compared with WT where a value of 1 means equal abundance. Italic protein names indicate an uncharacterized protein, which is annotated with a significant BLASTP homology search identifier.

To compare significant fold-change protein differences between the opaque mutants and WT, NSAF normalized spectral values (Supplementary Table S7) were used (Zybailov *et al.*, 2006). Twenty proteins were identified with a *P* value of <0.01 and fold changes either >2 or <0.5 compared with WT (Fig. 5). Clustering analysis, which determined the order of genotypes left to right in Fig. 5 for the evaluated significant proteins, revealed similar results to the PCA. *o1* and *fl1* clustered separately from the other mutants, and the UPR opaque mutants (*fl2*, *Mc*, and *DeB30*) were the most similar to each another. *DeB30* and *o2* were the most dissimilar from *o1* and *fl1* by this method of clustering. Only two of the 20 significant proteins were uncharacterized and had no domain-of-function information, but the majority of proteins listed were for those with higher accumulation (greater than 2-fold abundance) compared with WT.

The most general increase in protein abundance is indicated in the first five proteins listed but was not dramatically changed in *o1*. These proteins included adenosylhomocysteinase, cytosolic glucose-6-phosphate isomerase, nucleoside diphosphate kinase, grx_C2.2-glutaredoxin subgroup I, and a cyanate hydratase. All of the previously listed proteins can be grouped into general indicators of cellular stress. Adenosylhomocysteinase is involved in ethylene biosynthesis (Young and Gallie, 2000). A common increase in this protein in opaque mutants could imply a generalized increase in ethylene signaling, which could interfere with normal endosperm maturation, perhaps through accelerated programmed cell death.

Nucleoside diphosphate kinase provides fuel for membrane remodeling by generating of GTP from ATP (Boissan *et al.*, 2014). This was the only significantly increased protein across all samples and was not identified using the ISA. Glutaredoxin is involved in the glutathione–ascorbate cycle and has previously been shown to be increased in stress conditions (Yang *et al.*, 2014). Similarly, under sustained stress conditions in Arabidopsis, it has been shown that cyanate hydratase gene expression is increased (Qian *et al.*, 2011). An additional stress protein, B6TIK3, was found to have 2-fold higher accumulation in *DeB30*.

Two significantly increased proteins included Q6T5M1 (19kDa α-zein) and Q00LN5 (*Mucronate*-specific 16kDa γ-zein), which were enriched in the non-zein fraction. The 19kDa α-zein was significantly increased in *o1* (3.1-fold), *fl1* (2.6-fold), and to a lesser extent *DeB30* (1.2-fold). This may indicate an increased accumulation of α-zein outside the confines of the hydrophobic protein body core in these mutants, which could increase its solubility and partitioning into the non-zein fraction. In *DeB30*, this increase represents the mutant, unprocessed 19kDa α-zein, which remains attached on the periphery of the protein body. This is supported, since the peptides identified showed coverage overlap with the signal peptide in the case of *DeB30* but not in the 19kDa α-zeins, which were increased in *o1* and *fl1* (cleaved 19kDa α-zeins). As indicated by its presence in the top 50 most abundant proteins and enrichment analysis, a significant increase (7.4-fold) was seen for the *Mucronate*-specific 16kDa γ-zein (Q00LN5). This increase was unique to the *Mc* mutant because the *Mc* mutation causes a frameshift as a result of a 38bp deletion in the 16kDa γ-zein gene (Kim *et al.*, 2006). The mutant protein is a zein/non-zein chimera with a shorter, nonsense C-terminal sequence.

Other significant proteins with differential abundance compared with WT included those involved in starch metabolism such as the cluster of glucose-1-phosphate adenylyltransferase (Brittle-2), an additional cluster of glucose-1-phosphate adenylyltransferase (Sh2), and β-amylase. Sh2 (J7H390) had a general lower abundance in all mutants except for *o1*, which had a slight increase. Brittle-2 (Q947C0) was at higher abundance specifically in *o1*, while β-amylase was specifically increased in *fl1*. *fl1* also had a specific high accumulation for K7U1M0 and B4G218, which are not functionally annotated proteins. K7U1M0 has 100% sequence identity with maize predicted l-gulonolactone oxidase-like protein and is increased 2.5-fold over WT and all other opaque mutants. l-Gulonolactone oxidase is involved in vitamin C synthesis, which is a major antioxidant for cellular stress in plants (Venkatesh and Park, 2014). B4G218 has high sequence identity (84%) with foxtail millet desiccation-related PCC13-62-like protein and was increased 2.1-fold over all other samples. Desiccation-related PCC13-62 is a stress response protein that is strongly induced by abscisic acid (ABA) (Piatkowski *et al.*, 1990).

Flower-specific γ-thionin was represented twice as a significant protein derived from two separate genes (B6SJS8 and B6SJE6). γ-Thionins are defense-related proteins and contain defensin-A like motifs (Pelegrini and Franco, 2005). This defense-related protein had a large difference in abundance between the two UniProt accession identifiers. B6SJS8 had a higher abundance in *o1* and was slightly lower in *DeB30* and *Mc*, while *fl1*, *fl2*, and *o2* showed only a slight increase for this protein. B6SJE6 was of variably lower abundance in all mutants.

The remaining proteins had differential abundance in all mutants but clustered between similar groups of opaques. The 5a2 protein is annotated as a putative seed storage protein (B4FFB8), while low-molecular-weight cysteine-rich protein LCR70 (B6SMX5) is involved in the defense response. *fl1* and *o1* had a decrease in B4FFB8 and B6SMX5, which were increased in *DeB30* and *o2*. The translational machinery was also differentially abundant across the opaque mutants. *o2*, *Mc*, and *DeB30* all had an increase in B4FNT1, cluster of elongation factor 1-δ, which was lower in *fl2* and *o1*. Proteins that are induced by ABA were also represented with significant fold changes, which could indicate premature programmed cell death. B6T8E4 is an embryonic abundant protein 1 (EMP1), which seemed to be specific to *fl2*, showing a higher accumulation. EMP1 is strongly induced by ABA and in rice may function as a cytoplasm protectant during desiccation (Litts *et al.*, 1992). B6UI10 is the senescence-associated protein DIN1, which accumulated at higher levels in *o2* and at a lower abundance in *fl2* and *o1*, and is also induced by ABA (Oh *et al.*, 1996).

The second major cluster of proteins was specific to proteome rebalancing and thus these were increased in *o2* but not in *fl1* and *o1*. These selected proteins had a higher than average lysine content and most likely contribute to the overall qualitative increase of lysine in *o2*.

One lysine-rich protein that is well known to correlate with increased lysine content in *o2* is EF1-α (Habben *et al.*, 1995; Sun *et al.*, 1997). Surprisingly, EF1-α protein was decreased in *o2* and *fl2* in the current data. This likely results from partitioning of EF1-α into the zein fraction, since it is known to be tightly associated with the cytoskeletal network surrounding the zein protein body (Clore *et al.*, 1996), and thus the non-zein fraction assayed here may not accurately reflect the overall abundance of this protein in developing kernels.

## Discussion

There were several main objectives for this research. The first was to evaluate the contribution of the calculated lysine content in the most significantly changed proteins that is separate from the general increase in the whole non-zein proteome present in some but not all opaque mutants. The second objective was to screen for common proteins, apart from zeins, that may be necessary for vitreous endosperm formation during kernel maturation. A third objective was to appraise the value of shotgun proteomics to compare multiple isogenic kernel mutants.

In all opaque mutants studied, the molecular basis of the mutation has been identified along with the effect on protein body composition (Mertz *et al.*, 1964; Soave and Salamini, 1984; Schmidt *et al.*, 1990; Coleman *et al.*, 1997; Gillikin *et al.*, 1997; Kim *et al.*, 2003; Holding and Larkins, 2006; Kim *et al.*, 2006; Holding *et al.*, 2007; Wang *et al.*, 2012). However, the possible existence of common, zein-unrelated pathways or gene products linked to opaque endosperm formation in all mutants has remained unknown. Disruption in such a common mechanism could help to explain the opaque phenotype in mutants where quantitative or qualitative changes in zeins do not exist but cellular stress remains.

### Validity of the shotgun proteomic normalization approach to study opaque endosperm mutants

Label-free mass spectrometry is not considered quantitative because the composition of peptides can affect ionization efficiency. However, label-free proteomics can be used to identify relative protein abundance within a complex sample using well-characterized normalization methods such as absolute protein expression, NSAF, and exponentially modified protein abundance index (emPAI), and have been reviewed recently (Arike and Peil, 2014). The data was normalized using NSAF because it is the most reproducible for spectral count normalization across technical and biological replications compared with emPAI (McIlwain *et al.*, 2012). However, there are conflicting reports about which spectral count normalization algorithm is preferable (Trudgian *et al.*, 2011; Arike *et al.*, 2012; Ahrné *et al.*, 2013). The ability to draw conclusions on the quantitative abundance of individual proteins was supported by the comparison of transcript and protein abundance (Supplementary Table S2). Although transcriptional control stability is only partly responsible for protein abundance, in general there is a strong correlation between protein and transcript abundance changes, thus strengthening the assertion that protein abundance can be inferred using this method.

This study generated biological insights about endosperm opacity across a diverse set of opaque mutants through multiple statistically significant enrichment analyses. For example, the *Mucronate*-specific 16kDa α-zein protein, which was identified as one of the top 50 most abundant proteins, was enriched in the ISA uniquely in *Mc* and quantified using NSAF to have a 7.4-fold increase over not only WT but also all other opaque mutants. This particular zein protein example supports our label-free proteomic approach, subsequent normalization algorithm, and data analysis pipeline, and provides confidence in any non-zein protein changes reported.

Using LC-MS/MS shotgun proteomics, approximately 2700 total proteins were identified. The WT non-zein fraction had the most identified proteins (~2500), while all other mutants were less than or equal to 95% of this (Fig. 1). The total protein amount for each sample was standardized to 3 μg before liquid chromatography and indicated that proteome diversity may be reduced in mutants. The reduction of the mutants’ non-zein proteome may also be an indicator that only specific proteins are being increased or decreased, possibly below the detection limit, in order to compensate for the proposed cellular stress.

### Implications of changes to ER secretory pathway-related proteins on endosperm development

To give insight into general cellular stress that may occur in developing endosperm of all opaque mutants, and the possible impact on the secretory pathway, a general model is proposed (see Supplementary Fig. S2 at *JXB* online). The model is based upon results from the pathway and ISA enrichment and significant fold-change analysis in Figs 3 and 5 and Table 3. Forward trafficking after the ER is mediated by coat protein complex II (COPII), while reverse transport from the Golgi to the ER is mediated by COPI (Shima *et al.*, 1999; Barlowe, 2002). Each of the vesicle fusion steps between the Golgi and ER is mediated by vesicle transport machinery. At the surface of the membrane is a SNARE protein (soluble NSF attachment protein receptor), which cannot act alone in mediating proper fusion (Eakle *et al.*, 1988; Malhotra *et al.*, 1988). The two additional general factors needed are NSF and α-SNAP for proper integration to the designated cellular compartment (Eakle *et al.*, 1988; Malhotra *et al.*, 1988). *o1* was shown through ISA enrichment to have a lower abundance of α-SNAP, which may indicate that there are downstream trafficking problems, which in turn cause cellular stress through improper transport and delivery of essential developmental proteins to the Golgi or plasma membrane (Supplementary Fig. S2A).

*o2* may also have a trafficking problem that begins earlier in the secretory pathway than in *o1* (Supplementary Fig. S2B). ISA enrichment indicated a low abundance for Sec61-β protein, which is the central component for bidirectional transport to and from the ER (Zimmermann *et al.*, 2011). Sec61 is also a direct target for the O2 transcription factor (Li *et al.*, 2015). *o2* was determined through ISA enrichment to have unique protein associations with senescence-associated protein, which in Arabidopsis is strongly induced by phosphate starvation (Wu *et al.*, 2003). This possible phosphate starvation in the endosperm may also contribute to the lower enzyme activity of pyrophosphate-dependent phosphofructokinase, which has been reported previously in *o2* (Guo *et al.*, 2012).

The UPR mutants (*fl2*, *Mc*, and *DeB30*) have the highest number of shared proteins for the three-group enrichment, which are enriched for protein-folding and translation-related proteins. Key proteins increased are the cluster of protein disulfide isomerase (PDI), a KH domain-containing protein,a cluster of ribosomal protein, and a mitochondrial fission protein. A common UPR in the selected mutants was also highlighted. The increase in mitochondrial fission protein may serve to increase available ATP for the chaperones and HSPs increase compensation (Merrill and Strack, 2014). It is possible that the extent and extended duration of UPR may contribute directly to endosperm opacity (Supplementary Fig. S2C). Induced UPR in the ER lumen has been shown to produce reactive oxygen species (ROS), eventually inducing apoptosis (Malhotra *et al.*, 2008). It is possible that the normal, centrally emanating pattern of endosperm programmed cell death (Young and Gallie, 2000) could be disrupted or accelerated in opaque mutants exhibiting an elevated UPR. This could lead to opacity through early disruption of normal cellular metabolism in the endosperm. Figure S2D shows how protein folding affects ROS production. Upon ER import, the unfolded protein is immediately acted upon by PDI, which aids in the formation of properly folded proteins through disulfide bonds, which releases ROS in the process. Glutathione may also be consumed during the reduction of improper disulfide bonds in misfolded proteins (Malhotra *et al.*, 2008). This process seems to be affected in *fl1. fl1* is uniquely enriched for purple acid phosphatase, a glyoxylase family protein, and has significant fold changes for an l-gulonolactone oxidase (enzyme that produces ascorbate). All of the aforementioned proteins can contribute to the ascorbate–glutathione pool and act as antioxidants (Zhang *et al.*, 2008; Sanahuja *et al.*, 2013; Yang *et al.*, 2014). An increase in the ascorbate–glutathione pool supports the ROS scavenging capacity during ER stress (Ozgur *et al.*, 2014). This suggests that *fl1* may be able to lessen the extent of UPR through an increase of antioxidant proteins (Supplementary Fig. S2E).

### PCA of the internal structure of the non-zein proteome of opaque endosperm mutants

The underlying structure of each mutant’s non-zein proteome was shown using PCA of total proteins identified and also of proteins with significant fold changes compared with WT (Fig. 4A, B). It was apparent that *o2*, *Mc*, *fl2*, and *DeB30* have very similar non-zein proteomes and may indicate that such similarity is the result of their similarities in zein content changes. In *o1*, where zein content is not significantly affected, a clear separation from other samples was generated using total proteins identified and also using significantly changed proteins. However, it was interesting that in order to see separation of *fl1* from the other opaque mutants, significant fold-change proteins must be considered. *fl1* did show an overlap of total proteins identified with the mutants that had zein quantitative or qualitative changes but had a unique set of significant fold-change proteins.

### Enriched annotations and over-represented pathways in specific opaque mutants

The total non-zein proteome for opaque mutants is enriched for proteins involved in many diverse biological processes. One of the most common enrichments found in the functional annotation analysis was for proteins containing RNA recognition motifs, which include ribonucleoproteins (ribosomes) and transcription factors. Kernels were used at 20 d after pollination, when they are known to be at the peak of their general transcription and translation (Kodrzycki *et al.*, 1989). However, it was surprising that ribosomal proteins did not show significant fold changes in the mutants compared with WT, since *fl2*, *Mc*, and *Deb30* are known to have generalized translational repression as a result of ER stress and induced UPR (Hunter *et al.*, 2002). It was also surprising that eukaryotic elongation factor 1 δ (EF1-δ) was increased 2.1-, 1.6-, and 1.7-fold over WT in *DeB30*, *Mc*, and *o2*, respectively (Fig. 5). It is known that phosphorylating eIF2-α, which occurs upon activation of UPR, suppresses downstream transcription (Ron, 2002). This may indicate that, in these specific mutants, there is a compensation mechanism through EF1-δ for loss of transcription activity. The most enriched biological processes included protein transport, protein folding, proteolysis, and biosynthetic processes involving nitrogen or organic compounds. Since the majority of identified proteins were from membrane components, it is not surprising that protein transport was highlighted as an enriched process. Cellular stress has been identified previously in the endosperm of all opaque mutants (Hunter *et al.*, 2002) and plant cells can control their stress by either increasing quality control mechanisms or degrading the protein. Proteins involved in protein folding and proteolysis were two classes of enriched proteins.

### Amino acid compositional changes in the non-zein proteome as result of proteome rebalancing

When comparing non-zeins on a same protein weight basis, and not factoring in the known quantitative differences in the zein to non-zein ratio, we evaluated both up- and down-regulated proteins based on the changes of both lysine content and protein abundance in the lysine content analysis. We suggest that the discrete contributions of both increased and decreased proteins play a significant role in qualitative lysine increases in *o2* and *fl2*. The fact that the amino acid ratios for the top most abundant non-zeins is consistent with amino acid measurements for some amino acids in *o2* is likely a reflection of the large global increase in the proportion of all non-zein proteins. However, this trend only holds true for lysine in *fl2* and not for other amino acids, which may be strongly affected by zein accumulation. Interestingly, the decrease in zeins and increase in non-zeins was less significant in *fl2* than in *o2*. This suggests that qualitative differences in key lysine-rich proteins may play a greater role in lysine improvement in *fl2* than global increases in non-zeins. For *o1*, a mutant that neither increases lysine content nor rebalances the proteome, the *in silico*-calculated lysine content of the most abundant proteins was consistent with the previously measured total seed lysine content.

The lysine increase in *o2* has been most substantially attributed to the quantitative increase of non-zein proteins and general reduction of zeins. However, our data suggest that qualitative changes to the *o2* non-zein proteome are very significant in contributing to the overall increase in lysine. This qualitative change may also partly explain why some opaque mutants, which do not demonstrate a substantial increase in non-zein proteins, such as *fl2*, *Mc*, and *DeB30*, have an increase in lysine but not to the same extent as *o2*.

## Supplementary data

Supplementary data can be found at *JXB* online.

**Fig. S1.** Amino acid content analysis in non-zein proteins for *fl1*, *Mc*, and *DeB30* compared with WT.

**Fig. S2.** Diagram of key proteins affected in the opaque endosperm mutants and location in the ER secretory pathway.

**Table S1.** All proteins identified experiment-wide with raw spectral counts for each biological replication.

**Table S2.** Top significant proteins for lysine content analysis for *o2*, *fl2*, and *o1*, compared with WT.

**Table S3.** ISA unique protein enrichments for each genotype.

**Table S4.** ISA WT and *o1* protein enrichment.

**Table S5.** ISA UPR opaque mutants (*fl2*, *Mc*, and *DeB30*) protein enrichment.

**Table S6.** ISA six-group protein enrichment.

**Table S7.** NSAF normalized fold-change values and pairwise *t*-test corresponding *P* values compared with WT.

Supplementary Data
