# Supplementary material for: Proteomic profiling of maize opaque endosperm mutants reveals selective accumulation of lysine-enriched proteins
Source: J Exp Bot. 2015 Dec 27;67(5):1381–96. doi: 10.1093/jxb/erv532 (PMC4762381; doi:10.1093/jxb/erv532)
Supplement: Supplementary Data [file supp_67_5_1381__index.html]

Proteomic profiling of maize opaque endosperm mutants reveals selective accumulation of lysine-enriched proteins — Proteomic profiling of maize opaque endosperm mutants reveals selective accumulation of lysine-enriched proteins — Supplementary Data 

# Proteomic profiling of maize opaque endosperm mutants reveals selective accumulation of lysine-enriched proteins

## Supplementary Data

Data files

- supplementary\_figures\_S1\_S2.pdf - Supplementary Data
- supplementary\_tables\_S1\_S7.xlsx - Supplementary Data
