# Supplementary material for: Proteomic profiling of maize opaque endosperm mutants reveals selective accumulation of lysine-enriched proteins
Source: J Exp Bot. 2015 Dec 27;67(5):1381–96. doi: 10.1093/jxb/erv532 (PMC4762381; doi:10.1093/jxb/erv532)

Proteomic profiling of maize opaque endosperm mutants reveals selective accumulation of lysine-enriched proteins

Kyla Morton, Shangang Jia, Chi Zhang, and David Holding

Supplemental Figures

Supplemental Fig. S1. Amino acid content analysis in non-zein proteins for fl1, Mc and DeB30 compared to WT.

Supplemental Fig. S2. Diagram of key proteins affected in the opaque endosperm mutants and location in the ER secretory pathway.

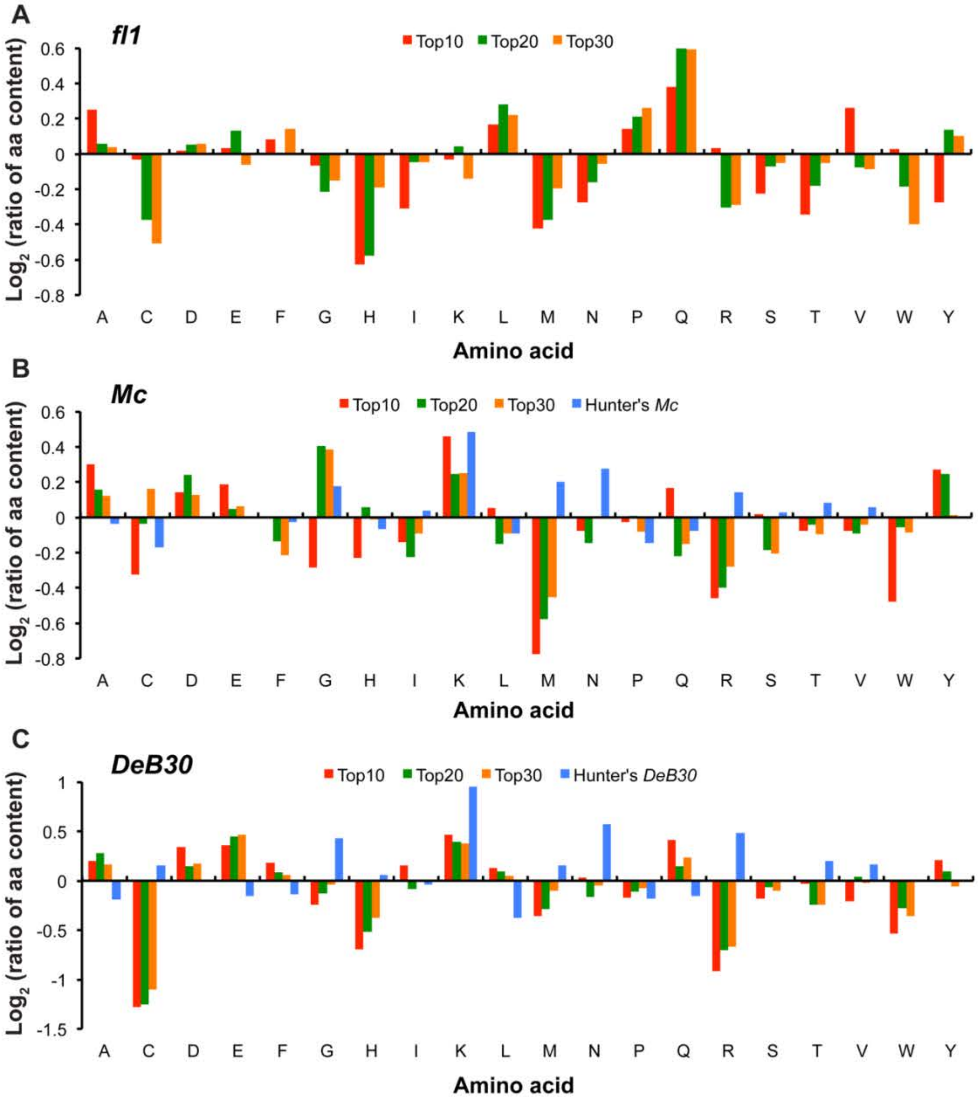

Supplemental Figure 1: Amino acid content analysis in non-zein proteins for *fl1*, *Mc* and *DeB30* compared to WT. Log_2 ratio of amino acid content was calculated using the ratio between average amino acid content of increased proteins to average amino acid content of decreased proteins, in comparison of genotypes and WT. The amino acid content data (blue, in percent protein, w/w) in *Mc* (B) and *DeB30* (C) from Hunter (2002). Amino acid abbreviations are shown in alphabetical order, and Hunter (2002) Glx was placed on both Glu (E) and Gln (Q).

Supplemental figure 2

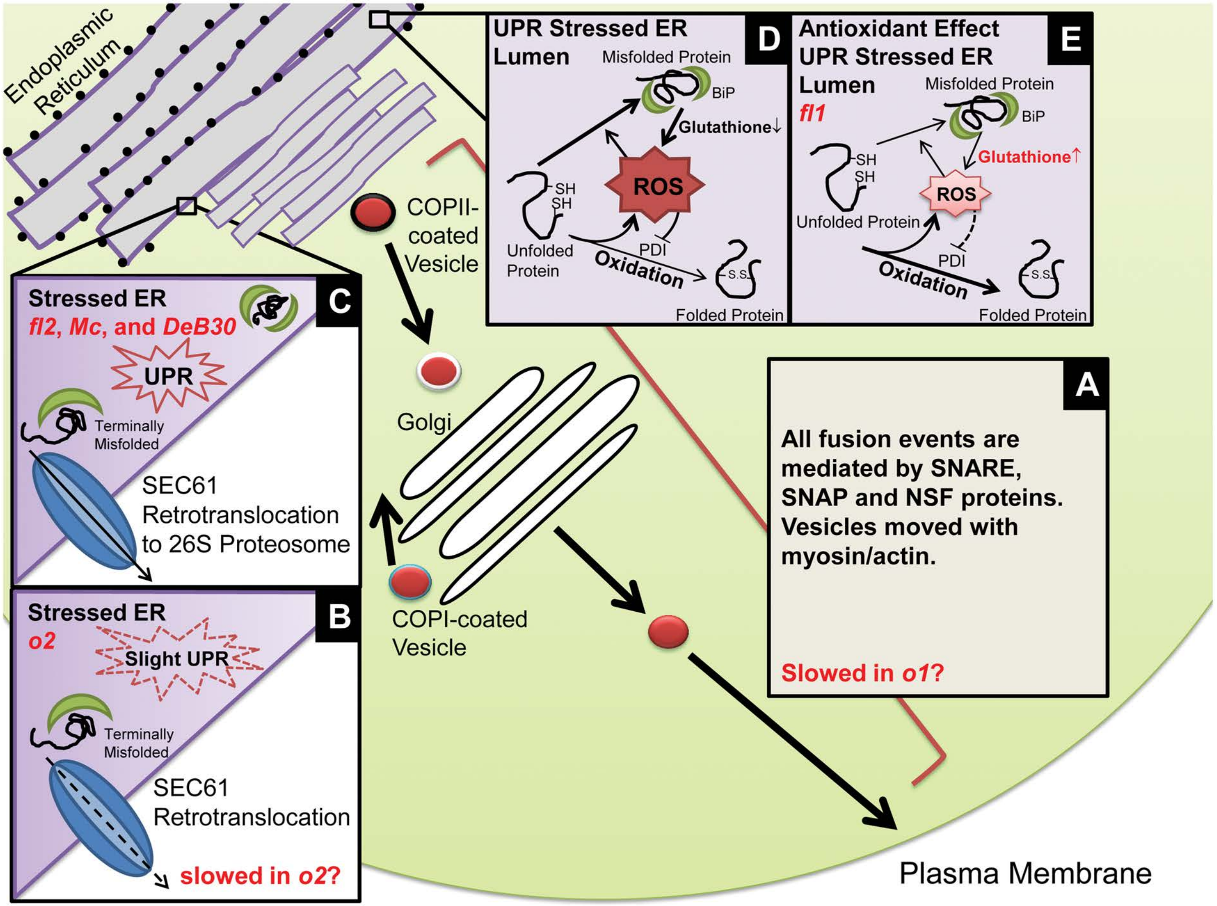

Supplement: Supplementary Data [file supp_erv532_supplementary_figures_S1_S2.pdf]
